# A Modular Biomimetic Preclinical Platform to Elucidate the Interaction Between Cancer Cells and the Bone Metastatic Niche

**DOI:** 10.3390/jfb16060220

**Published:** 2025-06-12

**Authors:** Claudia Cocchi, Massimiliano Dapporto, Ania Naila Guerrieri, Chiara Liverani, Marta Tavoni, Chiara Bellotti, Chiara Spadazzi, Anna Tampieri, Marco Gambarotti, Giacomo Miserocchi, Simone Sprio, Enrico Lucarelli, Michele Iafisco, Toni Ibrahim, Alessandro De Vita, Laura Mercatali

**Affiliations:** 1 Preclinic and Osteoncology Unit, Bioscience Laboratory, IRCCS Istituto Romagnolo per lo Studio dei Tumori (IRST) “Dino Amadori”, Via P. Maroncelli 40, 47014 Meldola, Italy; claudia.cocchi@irst.emr.it (C.C.); chiara.spadazzi@irst.emr.it (C.S.); giacomo.miserocchi@irst.emr.it (G.M.); alessandro.devita@irst.emr.it (A.D.V.); laura.mercatali@ior.it (L.M.); 2 Institute of Science, Technology and Sustainability for Ceramics (ISSMC), National Research Council (CNR), Via Granarolo 64, 48018 Faenza, Italy; massimiliano.dapporto@issmc.cnr.it (M.D.); marta.tavoni@issmc.cnr.it (M.T.); anna.tampieri@issmc.cnr.it (A.T.); simone.sprio@issmc.cnr.it (S.S.); michele.iafisco@issmc.cnr.it (M.I.); 3 Osteoncology, Bone and Soft Tissue Sarcomas and Innovative Therapies Unit, IRCCS Istituto Ortopedico Rizzoli, Via di Barbiano 1/10, 40136 Bologna, Italy; chiara.bellotti@ior.it (C.B.); enrico.lucarelli@ior.it (E.L.); toni.ibrahim@ior.it (T.I.); 4 Department of Pathology, IRCCS Istituto Ortopedico Rizzoli, 40136 Bologna, Italy; marco.gambarotti@ior.it

**Keywords:** bone metastasis, breast cancer, preclinical platform, tricalcium phosphate cements, osteoclasts, osteoblasts, patient-derived explants

## Abstract

Breast cancer (BC) frequently metastasizes to bone, leading to poor patient prognosis. The infiltration of cancer cells in bone impairs its homeostasis, triggering a pathological interaction between tumors and resident cells. Preclinical models able to mimic the bone microenvironment are needed to advance translational findings on BC mechanisms and treatments. We designed strontium-doped calcium phosphate cement to be employed for culturing cancer and bone cells and developed an in vitro bone metastasis model. The platform was established step by step, starting with the monoculture of cancer cells, mature osteoblasts (OBs) differentiated from mesenchymal stem cells, and mature osteoclasts (OCs) differentiated from Peripheral Blood Mononuclear Cells. The model was implemented with the co-culture of cancer cells with OBs or OCs, or the co-culture of OBs and OCs, allowing us to discriminate the interaction between the actors of the bone metastatic niche. The biomimetic material was further challenged with bone metastasis patient-derived material, showing good versatility and biocompatibility, suggesting its potential use as bone substitute. Overall, we developed a bone-mimicking model able to reproduce reciprocal interactions between cancer and bone cells in a biomimetic environment suitable for studying the biomolecular determinants of bone metastasis and, in the future, as a drug efficacy platform.

## 1. Introduction

Breast cancer (BC) is the most frequent cancer among women, and it is one of the most common cancer histotypes [1]. Bone is the most common site of systemic colonization by BC, with overt bone metastases (BM) often appearing decades after the diagnosis of the primary tumor [2,3]. Recent advances in the clinical management of BC patients have contributed to improving their prognosis, progression-free survival (PFS), and overall survival (OS) [3]. Nevertheless, bone relapse negatively impairs patient outcomes and quality of life, leading to the development of skeletal related events, such as pain, pathologic fractures, spinal compression, and hypercalcemia [4]. Therefore, understanding the metastatic process is crucial. The formation of BM involves a complex network of molecular signals that collectively affect the physiological bone remodeling. Indeed, cancer cells infiltrating the bone site can disrupt the physiological bone turnover by increasing osteoclasts (OCs) maturation both indirectly, by stimulating osteoblasts (OBs) to highly produce the Receptor Activator of Nuclear Factor Kappa B Ligand (RANKL) and reduce osteoprotegerin (OPG) release, and directly by expressing *RANKL* and other OC modulating factors. This results in an unbalanced maturation of OCs and subsequent pathological bone resorption. In turn, the degradation of the bone matrix leads to the release of growth factors (i.e., TGF-β) that augment BC-cell proliferation and bone colonization, establishing a vicious cycle. Cancer cells’ behavior in the bone site and their interactions with resident cells are affected by microenvironmental factors, including the stiffness and the high mineralization level of the bone extracellular matrix (ECM) [5,6,7,8,9].

Nowadays, the treatment of BM primarily focuses on palliating symptoms and halting disease progression. The median OS after BM diagnosis ranges between about 3 and 5 years for BC patients [10]. Therapeutic strategies for bone metastatic patients involve the use of integrated therapies, including bone-targeted agents. Among these, the bisphosphonate (i.e., zoledronic acid) and the monoclonal antibody anti-RANKL (denosumab) are the most used [11,12,13]. Recently, several pieces of evidence show that the characteristics of the tumor microenvironment can significantly influence drug response. For instance, due to the presence of an immunosuppressive microenvironment, the immune checkpoint inhibitors have demonstrated a lower response in the treatment of lung cancer-related bone lesions compared to other metastatic sites or to primary tumors [14].

Thus, representative in vitro models mimicking the native bone tumor microenvironment are warranted to improve preclinical drug screening and ultimately translate lead compounds into clinics. Over the last 20 years, a plethora of three-dimensional (3D) models have been established for this purpose. These models can be categorized as scaffold-free, such as spheroids or scaffold-based, where cells are cultured on either natural or synthetic supports. Scaffolds can be synthesized through different methodologies, such as 3D printing, electrospinning, or freeze-drying processes [15,16]. Biologic scaffolds are generally composed of native ECM proteins such as collagen, fibronectin, and tenascin, which favor adhesion and cellular interaction [17,18,19,20]. Additionally, tissue engineering has contributed to the production of bone-mimicking materials for the regeneration of bone defects [21,22,23]. Porous biomaterials based on nanocrystalline and ion-doped hydroxyapatite (HA) are widely regarded as one of the best options to develop bone scaffolds due to their high chemical and structural similarity with the mineral phase of bone tissue. This similarity is important to recreate a physiological bone-like microenvironment-promoting new bone formation [24,25]. Therefore, a biomaterial possessing these features is a valuable and effective candidate to reproduce the bone microenvironment for the in vitro evaluation of cellular mechanisms underlying bone disorders. In this work, we designed a strontium-doped apatitic bone cement (SrCPC) and developed a bone-mimicking model, leveraging its biomimetic composition and porous nanostructure. These characteristics were previously shown as relevant to promote osteogenic and osteo-integrative features by modulating the bone-cell fate towards more physiological bone turnover thanks to the presence of strontium [26]. Interestingly, SrCPCs were also associated with an osteo-inductive effect on mesenchymal stem-cell (MSC) differentiation and pre-OBs proliferation as well as an inhibitory effect on OCs activity [27].

In particular, we tested the ability of SrCPCs to support the differentiation of OCs from mononuclear precursors and OBs from human mesenchymal stem cells (h-MSCs) as well as cancer cells’ culture. Bone cements were also tested for the culture of patient-derived materials, including patient-derived explants (PDEs) and primary cultures. This approach allowed us to reproduce the mutual interactions between cancer and bone cells in a biomimetic environment that could be exploited as an advanced platform to investigate the cellular cross-talk of the bone metastatic niche.

## 2. Materials and Methods

### 2.1. Preparation of Sr-Doped α-Tricalcium Phosphate Cements (SrCPCs)

SrCPCs were prepared by mixing inorganic precursors and liquid components, as previously reported [26]. Briefly, a Sr-αTCP was prepared by dry-mixing calcium carbonate (CaCO_3_, Merck, Darmstadt, Germany), dicalcium phosphate dibasic anhydrous (CaHPO_4_, Merck, Darmstadt, Germany), and strontium carbonate (SrCO_3_, Merck, Darmstadt, Germany), followed by thermal treatment at 1400 °C for 1 h and rapid cooling. The stoichiometry was designed to obtain a final composition of Sr/(Ca + Sr) ≈ 2 mol%, previously associated with optimal biological outcomes [27]. Such a powder was milled by planetary mono mill (Pulverisette 6 classic line, Fritsch, Fellbach, Germany) for 50 min at 400 rpm using a zirconia jar with 5 mm diameter grinding media.

The liquid component of the paste was made of aqueous solutions of 5 wt% disodium hydrogen phosphate dihydrate (Na_2_HPO_4_∙2H_2_O, Fluka, Morris Plains, NJ, USA) and 2 wt% sodium alginate (Alginic Acid Sodium Salt from Brown Algae, Sigma-Aldrich, St. Louis, MO, USA). Finally, appropriate amounts of powder and liquid, according to liquid-to-powder (LP) ratio equal to 0.6, were mixed using a high-energy planetary shear-mixer (Thinky Mixer ARE-500, Thinky, Tokyo, Japan) at 1000 rpm for 90 s. The disk samples for biological testing were obtained after hardening in Teflon molds (diameter = 10 mm; height = 2 mm) for 30 min and then immersed in Hanks’ balanced salt solution at 37 °C for 3 days.

### 2.2. Characterization of SrCPCs

The crystallographic composition of the cements was investigated by X-ray diffraction (XRD) by using a D8 Advance diffractometer (Bruker, Karlsruhe, Germany) with CuKα radiation, 2θ range 10–80, scan step 0.02. The amount of α-tricalcium phosphate (αTCP) and HA crystalline phases was quantified by JCPDS file 029-0359 and 009-0432, respectively. The phase quantification was carried out by full-profile Rietveld refinement by using the software TOPAS v.5, Bruker, Karlsruhe, Germany, and the space group n. 176, P63/m, which is typically used to describe the apatite structure.

The initial and final setting times of the cement formulations were monitored by Gillmore needles according to standard ASTM C266-99. The chemical composition of bone cements, particularly oriented to the bone-mimicking chemical composition (Calcium, Phosphorus, and Strontium ions), was evaluated after 72 h in a thermostatic bath at 37 °C by inductively coupled plasma optical emission spectroscopy (5100 ICP-OES, Agilent Technologies, Santa Clara, CA, USA). The samples involved the dissolution of 20 mg of powders into 2 mL of nitric acid (HNO_3_, Sigma-Aldrich), followed by dilution to 100 mL with bi-distilled water. Three specimens were tested.

### 2.3. Sterilization and Preconditioning of SrCPCs

SrCPCs were sterilized in ethanol 70% for 1 h, washed three times in phosphate-buffered saline (PBS 1x), and put under germicidal UV-C radiation 200–280 nm for 1 h [28,29,30]. After sterilization, the cements were conditioned for 72 h in culture medium to permit the release of toxic components adsorbed from the materials during the synthesis process. After this preincubation, the cells were seeded.

### 2.4. In Vitro Culturing of BC Cells

The experiments were performed on MDA-MB-231, a triple negative BC cell line obtained from the American Type Culture Collection (ATCC, Rockville, MD, USA), with passage numbers around 100. The cells were routinely cultured as a monolayer in 75-cm^2^ flasks at 37 °C in α-MEM medium (PAA, Piscataway, NJ, USA) supplemented with 1% Penicillin/Streptomycin, 1% glutamine (PAA), and 10% fetal bovine serum (FBS) in a 5% CO_2_ atmosphere. The cancer cells were cultured in the same culture medium reported, firstly at the density of 1.5 × 10^4^ cells on SrCPCs and in 2D standard conditions. For the seeding on SrCPCs, the cells were resuspended in 15 µL of culture medium and put in a single drop in the center of the SrCPC, left 1 h in the incubator in order to allow the cancer cells to attach on the surface, and then 5 mL of culture medium was gently added. The viability was analyzed through MTT assay (Sigma-Aldrich) at different time points (24 h, 72 h, and 7 days) to obtain a growth profile overtime according to the manufacturing protocol.

### 2.5. Osteoclasts Differentiation from Human Peripheral Blood Mononuclear Cells

Human OCs were obtained from the differentiation of PBMCs derived from buffy coats of healthy donors who gave written informed consent to take part in the study. The conditions for differentiation on 2D standard culture were already established [31]. Briefly, monocytes were isolated from buffy coats by Ficoll density gradient. After two washes in PBS, the cells were resuspended in complete α-MEM (LONZA, Basel, Switzerland). The cells were counted and plated at a concentration of 1.5 × 10^6^ PBMCs in the 24-well plate and on the SrCPCs. After 4 h, the medium was removed and differentiation into OCs was induced by adding α-MEM supplemented with 20 ng/mL of Monocyte colony stimulating factor (M-CSF) (Cod. 300-25; Peprotech, Rocky Hill, NJ, USA). From day 7 of culture, 20 ng/mL of RANKL (Cod. 310-01; Peprotech, Rocky Hill, NJ, USA) was added with M-CSF. The medium was changed every 3 days. The negative control of OCs’ differentiation was PBMC-seeded at the same cell culture concentration and cultured with α-MEM without growth factors. After 14 days, the cells were fixed by incubation in 4% paraformaldehyde (PFA; Cod. 157-4; Polyscience, Niles, IL, USA) for 15 min at room temperature.

For 2D culture, the mature OCs were stained for tartrate resistant acid phosphatase (Cod. 3879A; TRAP kit, Sigma-Aldrich, Steinheim, Germany). Nuclei were counterstained with hematoxylin (TRAP kit). Cells with more than 4 nuclei and positive to TRAP staining were defined as OC-like cells. Images of differentiated cells were acquired at different magnifications using NIS-Element D 4.10.01 64-bit software (Nikon Corporation, Tokyo, Japan).

### 2.6. Osteoblast Differentiation from Human Mesenchymal Stem Cells

We optimized the osteoblastic differentiation protocol of hMSC (derived from human embryonic stem cells—Merck Millipore). The differentiation medium was composed of αMEM (Minimum Essential Medium—Alpha MEM Eagle, Lonza), 10% FBS, 1% Penicillin/Streptomycin, 1% L-glutamine, and differentiation factors, including Dexamethasone 100 nM, β-glycerophosphate 10 mM, and ascorbic acid 200 μM, whose concentration was optimized during the study. The hMSC cells were seeded on tissue culture plates in 2D condition in a density of 3000 cells/cm^2^, while on the SrCPCs, in a density of 3 × 10^4^ cells per cement, as reported for cancer cells. The medium was changed every 3 days. The negative control of OBs’ differentiation was hMSC-seeded at the same cell culture concentration and cultured with α-MEM without growth factors. Fourteen days after the start of differentiation, the cells were fixed in 4% PFA for 15 min at room temperature.

### 2.7. Immunofluorescence Analysis

Immunofluorescence staining was performed to detect mature OCs and OBs differentiated on SrCPCs. OCs were stained with Phalloidin to detect F actin rings with anti-Tartrate Resistant Acid Phosphatase (anti-TRAP) and counterstained with DAPI solution to detect nuclei.

OBs were stained with Phalloidin, anti-Osteocalcin antibody, and DAPI. Both negative controls (non-induced cells) and positive controls (induced cells) were stained as follows.

Briefly, the cells were washed 3 times with PBS and fixed with 4% PFA for 15 min at room temperature, followed by permeabilization with a blocking solution (PBS 1x + BSA 1% + Triton X-100 0.3%) for 5 min. Then, in order to evaluate *TRAP* or *osteocalcin* expression, the cells were incubated for 2 h at room temperature with a primary anti-TRAP antibody (rabbit, ab185716-Abcam) diluted 1:500 in blocking solution or a primary anti-osteocalcin antibody (rabbit, ab198228-Abcam) diluted 1:200 in blocking solution. The cells were washed with PBS and incubated for 1 h with a secondary goat anti-rabbit-conjugated antibody diluted 1:500 in PBS 1x (Life Technologies) in dark conditions. For all staining procedures, the cells were then washed 3 times with PBS and counterstained with phalloidin stock solution (1:40 in PBS 1x) (Life Technologies, Foster City, CA, USA) and DAPI (1:150 in PBS 1x) for 30 min in dark conditions at room temperature. For the 2D samples, the cells were then mounted in ProLong gold (Life Technology). All the samples were examined by A1 laser confocal microscope (Nikon Corporation, Tokyo, Japan) and analyzed using the NIS Elements software AR 5.21.03 64-bit (Nikon Corporation, Tokyo, Japan).

Live/Dead^®^ Viability/Cytotoxicity Kit (Invitrogen, Waltham, MA, USA) was performed on MDA-MB-231 on SrCPCs on day 1 and day 7 after seeding according to the manufacturing protocol and analyzed with A1 laser confocal microscope.

### 2.8. Cell Fluorescence Quantification

All images were acquired with confocal microscopy at 20× of magnification. The fluorescent images were then analyzed using ImageJ software (Version 1.52a) by selecting one cell at a time in an image and measuring the area, integrated density, and mean gray value. Using the calculation for corrected total cell fluorescence (CTCF) = integrated density (area of selected cell × mean fluorescence of background readings), as described by McCloy et al. [32], the fluorescence intensity of each cell was calculated using Excel. For each image, 10 background areas were randomly selected and used to normalize against autofluorescence, and 30 cells per image were evaluated, 3 images for each condition. This resulted in a mean fluorescence under each condition, which was then used for statistical analyses. Also, the area of each cell was recorded and analyzed.

### 2.9. Culture of Ex Vivo Material from Bone Metastatic Patients on SrCPCs

PDEs were obtained from 2 patients affected by BC-BM who underwent surgery maximum 48 ± 24 h before the processing. In this time frame, the tissues were vacuum-packaged and stored at 4 °C at the Department of Pathology. Tumor (Patient 1) and healthy peritumoral tissue (Patient 2) portions were selected by an expert pathologist. The tissue was washed once in PBS and manually cut into 3 mm^3^ samples obtaining PDEs. The samples were frozen in FBS (Life Technologies) + 10% DMSO (Sigma-Aldrich) solution and stored in liquid nitrogen until the start of the experiment. PDEs were cultured in ultra-low attachment 6-well plates, in DMEM (Dulbecco’s Modified Eagle’s Medium, Euroclone, Pero, Italy) 4.5 g/L glucose supplemented with 20% non-heat treated FBS, and 1% L-glutamine and 1% Penicillin/Streptomycin/amphotericin B (supplements from Life Technologies). A primary cell line, BMET-3, derived from the bone metastatic lesion of another patient affected by BC bone metastasis and subjected to surgical intervention was isolated. As for PDEs, the time from surgery to processing was 48 ± 24 h. The samples were frozen in FBS (Life Technologies) + 10% DMSO (Sigma-Aldrich) solution and stored in liquid nitrogen until enzymatic digestion. Tissue digestion was performed as described elsewhere [33,34]. BMET-3 cells were used between passage 3 and 5 for each experiment and cultured in DMEM 4.5 g/L glucose supplemented with 20% non-heat treated FBS and 1% Penicillin/Streptomycin/amphotericin B and 1% L-glutamine. Three different cellular seeding densities on cements were tested to define the best one for the analyses, and then it was set at 4.5 × 10^4^ cells per cement or per 48 well-plates (same surface area as SrCPCs).

Both PDEs and the primary cell culture were maintained at 37 °C and 5% CO_2_ in a humidified incubator.

The research protocol was approved by the IRCCS Istituto Romagnolo per lo Studio dei Tumori (IRST) Institutional review board and the Local Ethics Committees (AVEC and CEROM). The Ethical Committee approval number is PG N. 0012825 of 12/11/2018. The procedures were in accordance with the Helsinki Declaration of 1975, as revised in 2008.

### 2.10. Viability Assay of the Ex Vivo Models

For both PDEs and BMET-3 cells, we performed viability analyses through Presto Blue^TM^ reagent (Invitrogen), following protocol’s instruction. In brief, Presto blue was added at 10% in complete culture medium and incubated for 4 h (BMET-3 cells) or 5 h (PDEs) at 37 °C in the incubator. After the incubation, 100 µL of culture medium was transferred in a 96-well total black plate, and absorbance was read and expressed as a ratio between λ 530 and 590 nm (microplate reader Synergy HT, Bio-Tek Instruments Inc.). The analyses were carried out on days 1, 3, and 7 after seeding. The viability data are shown as a fold-change ratio on the absorbance of the control sample (PDE only) on day 1 for PDEs, while for BMET-3, the data are shown as the ratio on day 1 of the measurement for each seeding density condition.

On day 7, to better visualize BMET-3 cell spatial organization on cements, we performed Live/Dead staining (Molecular Probes, Invitrogen). Each cement was incubated for 10 min at 37 °C with 500 µL of staining solution composed of 2.5 µM Calcein AM and 5 µM of ethidium homodimer-1 in PBS. The SrCPCs were then washed with fresh PBS, and pictures were taken using an inverted Nikon Eclipse TE2000-U microscope equipped with a calibrated Nikon DS-Vi1- U3 CCD camera.

### 2.11. Evaluation of Cellular Necrosis of Ex Vivo Models

For both PDEs and BMET-3 cells, we performed the analysis of cellular necrosis through the LDH-Glo™ Cytotoxicity Assay (Promega, Madison, WI, USA), following the manufacturer’s instructions. Briefly, 4 µL of culture medium of each experimental condition (the same wells used for viability evaluation) were collected, diluted in 196 µL of LDH storage buffer, and stored at −20 °C until quantification. On the day of the test, all samples were diluted in the LDH storage buffer until 1:300 ratio was reached to minimize FBS-derived LDH’s interference, as mentioned in the protocol. Luminescence was recorded after 1 h of incubation using a microplate reader (Synergy HT, Bio-Tek Instruments Inc., Winooski, VT, USA). The data are shown as LDH U/mL, and were released and quantified in the culture medium of the same samples used for viability assay. No data normalization was performed.

### 2.12. Histology

On days 1, 3, and 7 after seeding, following the viability analysis, one sample for each experimental condition was washed with PBS and fixed in 4% formalin overnight at 4 °C. The day after the samples were processed for paraffin embedding, 4 µm thick histological sections were obtained by formalin-fixed, paraffin-embedded tumor samples and stained with hematoxylin and eosin in an automatic slides stainer. The evaluation of viable tissues was performed by an expert pathologist, examining the whole tumor section area. Viable tissue was calculated considering the percentage of stainable (viable) nuclei.

### 2.13. Gene Expression Analyses

#### 2.13.1. Procedure for Cell Culture Experiments

Total RNA from OCs and OBs seeded on SrCPCs or in 2D condition was isolated using TRIzol Reagent (Invitrogen, Carlsbad, CA, USA) following the manufacturer’s instructions. After a quantification protocol using NanoDrop 1000 Spectrophotometer (ThermoFisher Scientific, Waltham, MA, USA), two hundred and fifty nanograms of RNA were reverse-transcribed using the iScript cDNA Synthesis Kit (BioRad, Hercules, CA, USA). Real-time PCR was performed on the 7500 Real-time PCR System (Applied Biosystems, Foster City, CA, USA) using TaqMan gene expression assay mix (Applied Biosystems) or SYBR Green mix. The stably expressed endogenous *HPRT* gene was used as reference (F: 5′-AGA-CTT-TCG-TTT-CCT-TGG-TCA-GG-3′; R: 5′-GTG-TGG-CTT-ATA-TCC-AAC-ACT-TCG-3′; Hs99999909_m1). The following markers were analyzed in OCs: *ACP5* (acid phosphatase 5, tartrate resistant; Hs00356261_m1), *NFATC1* (Nuclear Factor of Activated T Cells 1; Hs00542675_m1), *JDP2* (Jun dimerization Protein 2; F: 5′-AGA-CTT-TGC-TTT-CCT-TGG-TCA-GG-3′; R: 5′-GTG-TGG-CTT-ATA-TCC-AAC-ACT-TCG-3′), *CTSK* (Cathepsin k; F: 5′-GCC-AGA-CAA-CAG-ATT-TCC-ATC-3′; R: 5′-CAG-AGC-AAA-GCT-CAC-CAG-AG-3′), and *CAII* (Anidrase carbonase II; Hs01070108_m1). The markers that were analyzed for OBs were the following: *ALPL* (Alkaline Phosphatase; Hs01029144_m1), *RUNX2* (Hs01047973_m1), and *COL1A1* (Collagen 1; Hs00164004_m1), OPG (Osteoprotegerin TNFRSF11B, Hs00900358_m1) (Life Technologies).

#### 2.13.2. Procedure for Ex Vivo Models

On days 3 and 7, one PDE per condition was flash-frozen in liquid nitrogen and stored in a liquid nitrogen tank until RNA extraction. RNA was extracted using the RNA/DNA/Protein Purification Plus Kit (Norgen Biotek Corp., Thorold, ON, Canada) following kit instructions. To obtain a homogenous powder, mortar and pestle were used, maintaining the samples frozen in liquid nitrogen.

For BMET-3 cells, on days 3 and 7, one cement per condition was immersed in 1 mL of TRIzol Reagent for 10 min, centrifuged at 1500 rpm for 5 min, and then stored at −20 °C until extraction, following the manufacturer’s protocol.

The obtained RNAs were quantified using the Denovix DS-11 spectrophotometer. Up to five hundred nanograms (when available) of RNA were retro-transcribed using the RT2 First Strand Kit (Qiagen, Hilden, Germany). One µL of the obtained cDNA was used to run Real-Time PCR analyses. Custom RT2 expression arrays (Qiagen) were designed, and the subsequent primers were spotted onto 100-well disks. Analyses were performed using the RotorGeneQ instrument and RT2 SYBR Green FAST Mastermix (Qiagen). All primers were designed by Qiagen, and the relative assay catalogs are listed in Table 1.

For both procedures, the number of transcripts was normalized to the endogenous reference genes and expressed as n-fold mRNA levels relative to a calibrator using a comparative threshold cycle (Ct) value method (ΔΔCt) [35].

### 2.14. Statistical Analyses

For the in vitro test, each experiment was repeated at least two times and with three technical replicates for each condition. The data are presented as mean ± SD. Student’s *t*-test was used as appropriate and accepted as significant at *p* < 0.05. The data were analyzed using GraphPad Prism 8.4.3.3.

For the ex vivo models and the primary cell culture, each experiment was performed in three biological and technical replicates for each condition, and for the PDEs model, the data were obtained from two different patients. The data are presented as mean ± SD and were analyzed using GraphPad Prism 8.4.3.3. Paired or unpaired Student’s *t*-test were performed as appropriate, and significance was accepted for a *p*-value < 0.05.

## 3. Results

### 3.1. Characterization of Strontium-Doped Apatitic Bone Cement (SrCPCs)

**Figure 1:**
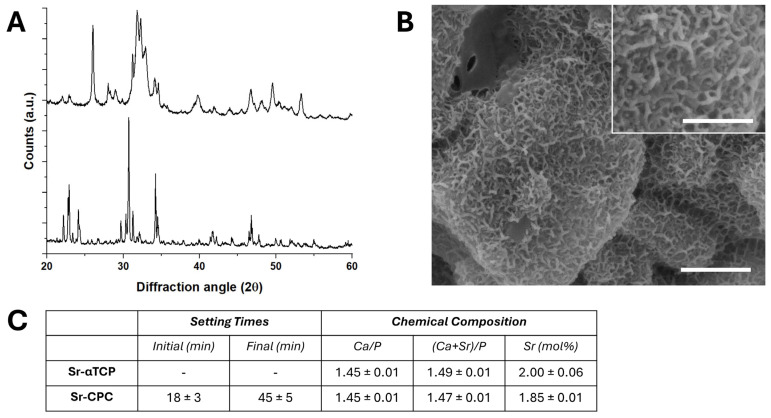
Characterization of SrCPCs. (**A**) XRD analysis of bone cements, inorganic precursor (**bottom**) and hardened cement (**top**). (**B**) ESEM micrographs of the surface of the samples (scale bar: 2 μm, inset scale bar: 200 nm). (**C**) Chemical composition of inorganic precursor and hardened cements with the respective setting times.

The mixing of the powder and liquid components resulted in handleable and fully extrudable cement formulations. The complete transformation of the SrCPCs precursor into Sr-doped HA was assessed by X-ray powder diffraction (XRD), revealing the absence of secondary phases at 72 h upon mixing (Figure 1A). The hardening times revealed that a structural rearrangement of the HA crystals within 45 ± 5 min, while the needle-like microstructure of the surfaces also confirmed the dissolution of the precursor powder, followed by the re-precipitation into apatitic crystals (Figure 1B). Furthermore, the chemical analysis confirmed the presence of a nominal amount of strontium, both in the precursors and the final cement (Figure 1C). On this basis, SrCPC disk-shaped samples for biological testing were prepared 72 h after mixing.

### 3.2. Culture of BC Cells on SrCPCs

**Figure 2:**
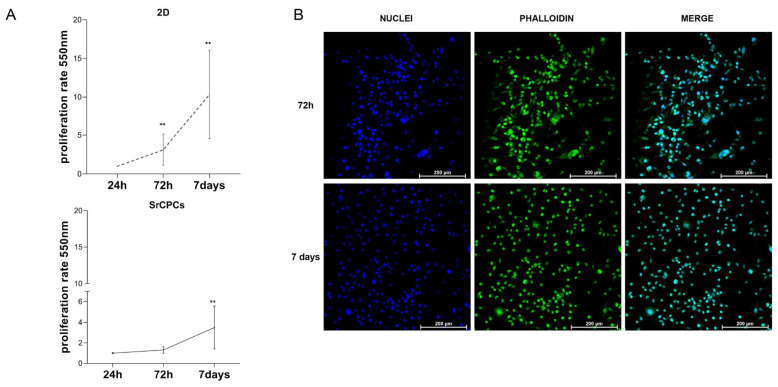
Breast cancer cell monoculture on SrCPCs. (**A**) Growth replicate curves of BC cell line MDA-MB-231 from day 1 to day 7 in 2D condition (**top**) and on SrCPCs (**bottom**). (**B**) Confocal microscopy images of MDA-MB-231 on SrCPCs at 20× magnification on day 3 (72 h) and day 7 of culture. The cells are stained with DAPI (blue—first column) and Phalloidin AlexaFluor488 (FITC, green—second column). In the third column, the merge of the staining is shown. All experiments were performed in biological duplicates, each with three technical replicates. The data are expressed as Mean ± SD. ** *p* < 0.01.

The SrCPCs were cellularized with the triple negative mesenchymal-like BC cell line MDA-MB-231, a cell line that metastasizes to bone from bloodstream after intracardiac injection into murine models [36]. The cancer cells’ growth profile on SrCPCs was firstly assessed and compared with that obtained on 2D standard tissue culture plates. A significant increase in cell number was observed at 72 h and 7 days of culture (*p* < 0.01), but the proliferation rate was lower compared to the 2D monolayer condition (Figure 2A). Under confocal microscopy, the MDA-MB-231 cells exhibited a mesenchymal-like phenotype with disorganized aspects and were homogeneously distributed over the entire surface of SrCPCs (Figure 2B). The Live/Dead staining revealed that SrCPCs were biocompatible for cancer cells (green cells), in absence of toxicity, and detected after 24 h and 7 days upon cancer cell seeding (red cells) (Figure S1).

### 3.3. OCs Differentiation on SrCPCs

**Figure 3:**
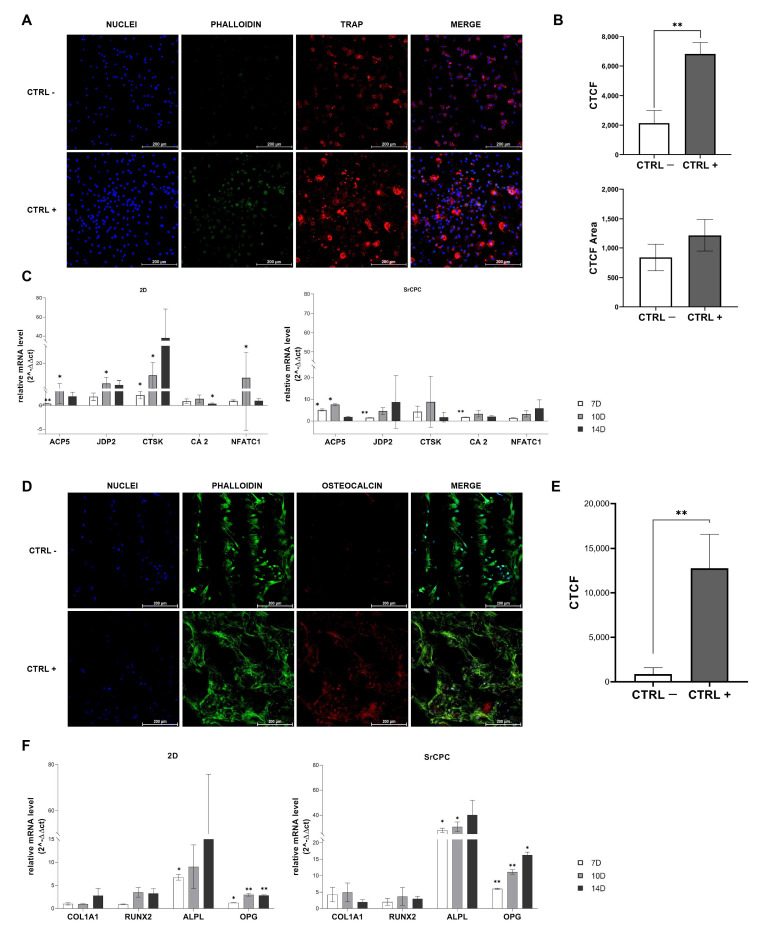
Differentiation of bone cells on SrCPCs. (**A**) Confocal microscopy images of PBMCs on day 14 of culture in differentiation to OCs at 20× magnification. The three columns refer to the DAPI staining of nuclei, the TRITC staining for F-actin (Phalloidin), and the FITC staining to detect TRAP, a marker of mature OC. The stainings are merged in the fourth column. (**B**) Evaluations of the corrective total cell fluorescence (graph above) and the cell area (graph below) by Image J software. Thirty cells per images, only on SrCPCs, were analyzed for a total of ninety cells. The data are mean ± SD. ** *p* < 0.01. (**C**) Gene expression analyses were carried out by real-time PCR at different time points. Left: in 2D condition; right: on 3D SrCPCs. The reference sample was the negative control of OCs’ differentiation collected on day 7 of culture. 2^−∆∆Ct^ method was used for analysis. The data are mean ± SD. Statistical significance was performed with Student’s *t* test: * *p* < 0.05. ** *p* < 0.01. Biological replicates = 2; technical replicates = 3. (**D**) Confocal microscopy images of H-MSC on day 14 of culture in differentiation to OBs at 20× magnification. The three columns refer to the DAPI staining of nuclei, the FITC staining for F-actin, and the TRITC staining to detect Osteocalcin, a marker of mature OB. The stainings are merged in the fourth column. (**E**) Evaluation of corrective total cell fluorescence by Image J software. Thirty cells per image were analyzed for a total of ninety cells. The values were normalized with respect to CTRL−. The data are mean ± SD. ** *p* < 0.01. (**F**) Gene expression analyses were carried out by real-time PCR. The reference sample was the negative control of OBs’ differentiation, collected on day 7 of culture. The 2^−∆∆Ct^ method was used for analysis. The data are mean ± SD. Statistical significance was performed with Student’s *t* test: * *p* < 0.05. ** *p* < 0.01. Biological replicates = 2; technical replicates = 3.

OCs were obtained by seeding PBMCs on SrCPCs and under differentiating factors (M-CSF and RANK-L). The presence of mature OCs was confirmed by immunofluorescence staining and confocal imaging (Figure 3A). In the positive control (CTRL+), we observed mature multinucleated OCs with at least four nuclei positive to the Tartrate Resistant Acid Phosphatase (TRAP) [31] and showing a ring-shaped cytoskeletal organization. Mature cells, namely PBMCs cultured without differentiating growth factors (CTRL−), were not observed in the negative control (Figure 3A).

Correction total cell fluorescence (CTCF) was evaluated from TRAP fluorescence intensity. The cells treated with osteoclastogenesis growth factors showed a significantly higher CTCF compared to the negative control (*p* = 0.015), confirming the successful differentiation of OCs (Figure 3B, top). As cell area is a surrogate parameter of OCs maturation [31], we measured this parameter in negative- and positive-control samples but without observing a statistical significance (Figure 3B, bottom).

OCs’ maturation was also confirmed by the gene expression analyses of some of the most relevant osteoclastogenesis markers. The results displayed an increased overtime relative expression level of *JDP2* and *CAII* (day 7; *p* = 0.008; *p* = 0.0055, respectively) compared to the negative controls. In addition, *CTSK* and *ACP5* (*TRAP*) increased till day 10 (*ACP5 p* = 0.012), after which they decreased in the last time point (Figure 3C, right). Gene expression analyses of 2D samples showed the same trend for each marker, reaching higher absolute values when PBMCs were cultured on 2D plastic monolayers (Figure 3C, left).

### 3.4. OBs Differentiation on SrCPCs

OBs obtained from the differentiation of commercial hMSCs were also included in the model. Osteoblastogenesis on SrCPCs was evaluated by confocal imaging (Figure 3D). Cells changed morphology to become spindle-like and positive for the osteoblastogenesis marker osteocalcin in the CTRL+ compared to the CTRL−. CTCF was evaluated from the osteocalcin fluorescence intensity: we observed that cells treated with differentiating growth factors showed a significantly higher CTCF compared to the negative control (*p* = 0.0396), confirming the differentiation of OBs (Figure 3E).

Differentiation in 2D and on SrCPCs was also confirmed by the gene expression analysis of *ALPL*, *COL1A1*, *OPG*, and *RUNX2* (Figure 3F). For all the analyzed markers, an increase in the relative expression levels was observed over time compared to the negative control in cells grown on SrCPCs (Figure 3F, right). In the samples grown on 2D standard culture, the differentiation markers reached higher values of expression compared to those grown on SrCPCs for *RUNX2* and *COL1A1* (Figure 3F, left). The upregulation of *OPG* and *ALPL* can be correlated with the effective OB maturation that is responsible for the bone turnover.

### 3.5. Cancer Cells and Bone Cells Co-Cultures

**Figure 4:**
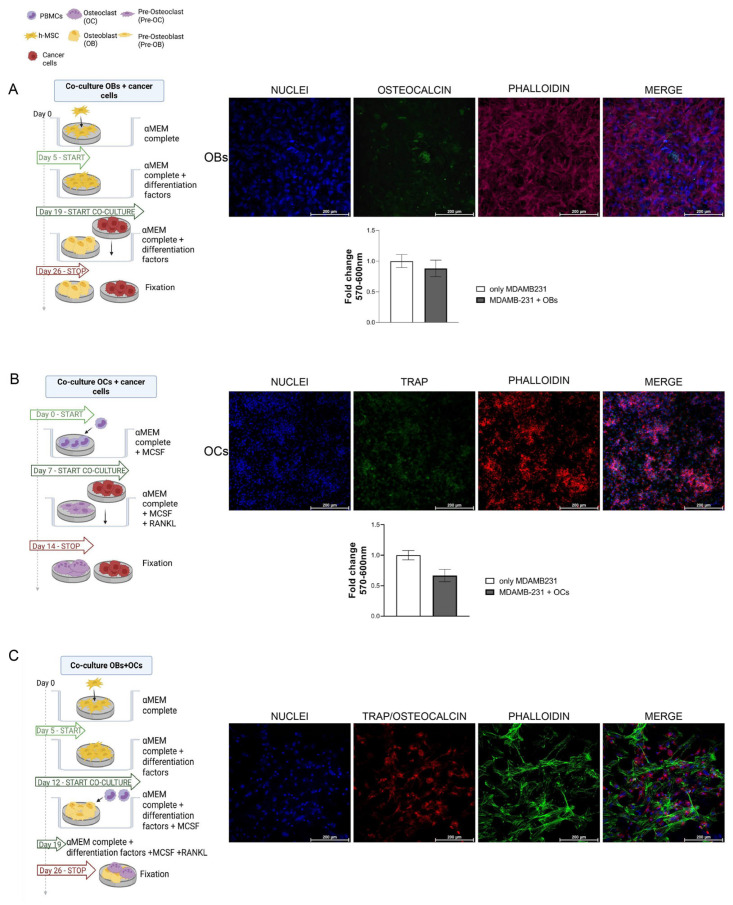
Co-cultures on SrCPCs. (**A**) Scheme of co-culture of OBs and cancer cells. Confocal microscopy images of h-MSC in differentiation to osteoblasts at 20× on day 26 of culture. The three columns refer to the DAPI staining of nuclei, the TRITC staining for F-actin (Phalloidin), and the FITC staining to detect Osteocalcin, a marker of mature osteoblast. The stainings are merged in the fourth column. MTT proliferation cancer cell graph of two biological replicates in co-culture or alone. Mean ± SD. (**B**) Scheme of co-culture of OCs and cancer cells. Confocal microscopy images of PBMCs in differentiation to OCs at 20× on day 14 of culture. The three columns refer to the DAPI staining of nuclei, the TRITC staining for F-actin (Phalloidin), and FITC staining to detect TRAP, a marker of mature OC. The stainings are merged in the fourth column. MTT proliferation cancer cell graph of two biological replicates in co-culture or alone. Mean ± SD. (**C**) Scheme of co-culture of OBs and OCs. Confocal microscopy images of h-MSC and PBMCs in differentiation to OBs and OC, respectively, at 20× on day 26 of culture. The three columns refer to the DAPI staining of nuclei, the TRITC staining for F-actin (Phalloidin), and the FITC staining to detect Osteocalcin and TRAP. The stainings are merged in the fourth column. Created in https://BioRender.com (accessed on 5 March 2025).

We then optimized co-cultures of bone cells with cancer cells on SrCPCs, including (a) cancer cells with hMSCs toward OBs’ differentiation (Figure 4A), (b) cancer cells cultured with PBMCs towards OCs differentiation (Figure 4B), and (c) PBMCs and hMSCs in differentiation to OCs and OBs (Figure 4C), respectively. For bone cells’ co-culture, PBMCs and hMSCs were seeded on the same SrCPC in a culture medium supplemented with OB- and OC-specific differentiating factors. For cancer cell OB and cancer cell OC co-cultures, each cell type was cultured on dedicated specimen sharing the culture medium (Figure 4A,B). In particular, the SrCPC seeded with cancer cells was added to the same well with the one where bone cells were differentiating on day 7 of the experiments of OC co-culture and on day 19 for OB co-culture (see the timeline experimental schedule in Figure 4). The co-cultures were stopped at different timepoints, as reported in Figure 4, in order to allow the complete maturation of bone cells. In either of the co-culture condition of cancer cells with OCs or OBs, there was not a significant difference in cancer cells growth, as evaluated with MTT assay. Healthy cells were able to differentiate both towards OCs and OBs in co-culture conditions. As shown with confocal microscopy images, all cell types appeared in a good fashion and covered the entire surface of the SrCPCs.

### 3.6. Ex Vivo Validation of the Biocompatibility of SrCPCs

**Figure 5:**
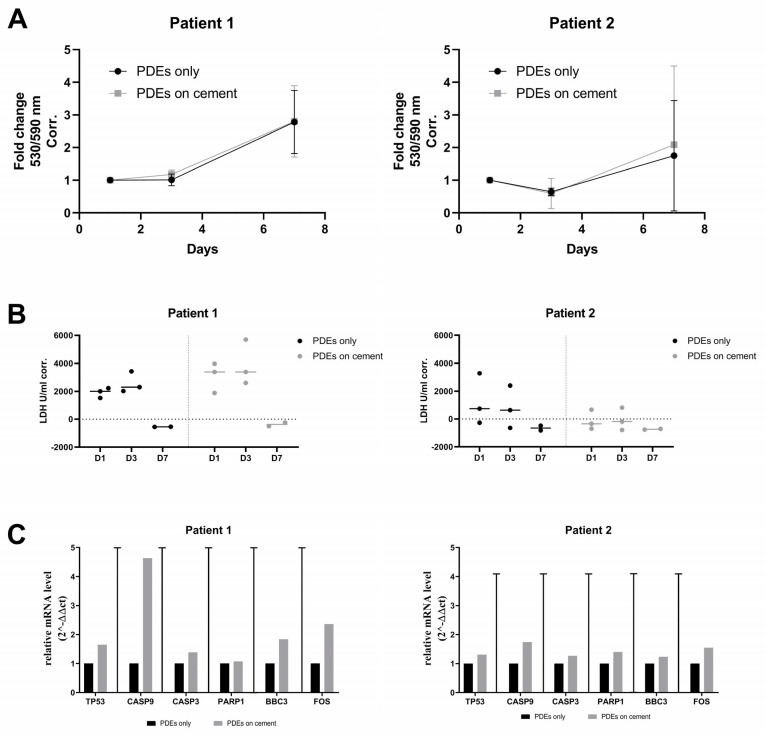
Ex vivo validation of SrCPCs’ effects on PDEs. (**A**) Viability assay using Presto Blue^®^ reagent performed on days 1, 3, and 7 after seeding. (**B**) Evaluation of cellular necrosis through quantification of the LDH released in culture medium on days 1, 3, and 7 after seeding. (**C**) The gene expression analyses were performed on total cellular RNA extracted from half of PDEs sacrificed for each time point (days 3 and 7 after seeding). The 2^−∆∆Ct^ method was used to analyze the relative expression of the target genes. The data were normalized on the expression of *GAPDH* housekeeping gene of PDEs only on day 3. Each experiment was composed of three PDEs per condition. One PDE per condition was sacrificed on day 3 or 7 after seeding and after viability/necrosis analyses and cut into two pieces used for RNA extraction or H&E stain (see Figure 6).

**Figure 6:**
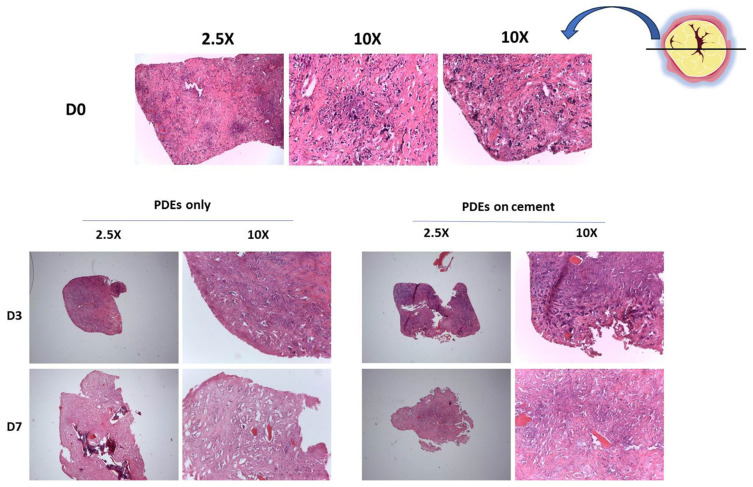
H&E staining showing good biocompatibility of SrCPCs on PDEs. (**Upper panel**): Representative pictures of the baseline tissue architecture and viability of Patient 1’s material before the culture set up (day 0). (**Bottom left panel**): Representative picture of PDEs only after 3 and 7 days of culture. (**Bottom right panel**): Representative picture of PDEs cultured on SrCPCs after 3 and 7 days of culture. All pictures were captured at the inner part of PDEs’ tissue samples.

For the ex vivo validation of the biocompatibility of SrCPCs, we performed viability, necrosis, and gene expression analyses both on metastatic and healthy peritumoral tissues as patient-derived explants (PDEs) as well as on the primary cell line BMET-3. The results obtained on two PDEs batches showed a preserved viability and no difference in secreted LDH when the samples were cultured alone or in the presence of the SrCPCs (Figure 5A,B). A non-biologically relevant modulation of cellular apoptosis pathway was noticed with no statistical significance (Figure 5C). We also performed hematoxylin and eosin staining of Patient 1 samples to evaluate the internal structure of the PDEs over time in culture. At the basal level, the metastatic tumor tissue appeared vital with some areas of deterioration. On day 3 after seeding, the PDEs cultured on 2D standard cell culture had about 60% of vital tissue, comparable to those cultured on the SrCPCs (Figure 6). On day 7, all the samples had extensive necrotic regions, particularly in the tumor area (Figure 6). Additional images of superficial parts of the PDEs are presented in Figure S2.

**Figure 7:**
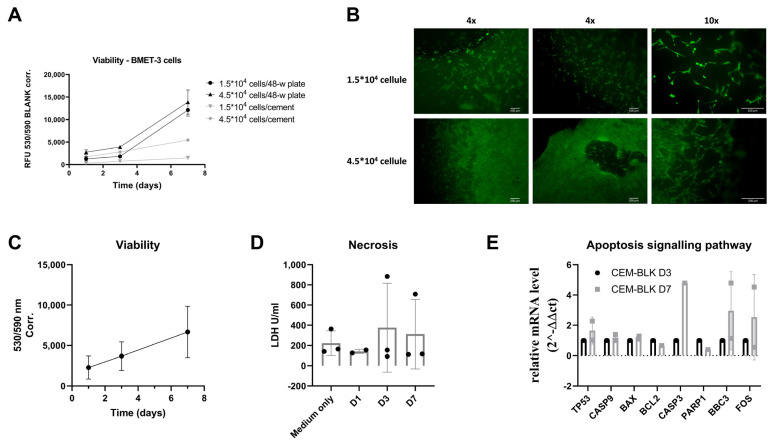
Ex vivo validation of SrCPCs effects on BMET-3 patient-derived cells. (**A**) Viability assay using Presto Blue^®^ reagent performed on days 1, 3, and 7 after the cells’ seeding on a 48-well plate or SrCPCs. (**B**) Representative images of a Live/Dead assay on day 7 after seeding cells on SrCPCs. Scale bar = 200 µm. (**C**) Viability assay using Presto Blue^®^ reagent performed on days 1, 3, and 7 after the cells’ seeding on SrCPCs. (**D**) Evaluation of cellular necrosis through quantification of the LDH released in culture medium on days 1, 3, and 7 after seeding on SrCPCs. (**E**) Gene expression analyses performed on total cellular RNA extracted from BMET-3 cells grown on one SrCPC sacrificed for each time point (Days 3 and 7 after seeding). The 2^−∆∆Ct^ method was used to analyze the relative expression of the target genes, and Student’s *t*-test was performed as appropriate without reaching statistical significance. The data were normalized on the expression of the *GAPDH* housekeeping gene on day 3. Each experiment was conducted in technical and biological triplicate, excluding non-measurable samples for gene expression analyses.

Similar experiments were conducted on the primary cell line BMET-3. We compared primary cells’ growth in 2D versus on SrCPCs and found a similar result to that shown above for MDA-MB-231 cells. Specifically, when seeded on SrCPCs, BMET-3 cells showed a slower proliferation than in 2D conditions (Figure 7A). In addition, the representative pictures of the Live/Dead staining performed on day 7 after seeding allowed us to observe a homogeneous distribution of BMET-3 cells through the SrCPCs’ surface and preserved viability (Figure 7B). Globally, primary cell viability tends to slightly increase over the 7 days of culture even on SrCPCs (Figure 7C). No relevant changes in LDH release were measured (Figure 7D). In parallel, a weak activation of the apoptosis pathway was detectable through gene expression analyses, which was neither statistically nor biologically significant (Figure 7E).

## 4. Discussion

Preclinical models of BM capable of comprehensively mimicking the metastatic niche could be outstandingly useful to understand the mechanism of BM formation and to develop new drugs that prevent the related destructive effects.

Modeling bone cancer requires careful consideration of key biophysical and biochemical parameters, including matrix stiffness, porosity, mineral composition, and fluid dynamics, which are all distinct in bone tissue compared to other organs. In particular, matrix rigidity has a well-documented impact on cancer cell invasion, metastasis, and tissue-specific tropism [37]. The currently available preclinical models reported in the literature involve the use of tissue engineering and the attempt to use human bone as a starting material [38,39,40].

Recent approaches have employed bone modeling that involves decellularizing native bone tissue via chemical, enzymatic, physical, or combination treatments. For example, Marinkovic et al. developed decellularized ECMs derived from bone marrow and adipose-derived stromal cells, which support the proliferation of both cancer and MSC, albeit showing differences in architecture and physical properties. The authors reported that ex vivo-derived native ECMs effectively recreate the specific microenvironments, or niches, of bone marrow and adipose-derived MSCs. [41]. An emerging alternative approach to model BM is 3D bioprinting, which enables precise control over scaffold architecture, stiffness, and ECM composition [42]. This technique supports cell viability, migration, and phenotypic maintenance, facilitating the generation of reproducible, bone-mimicking microenvironments. Zhu et al. studied the interaction between human fetal osteoblasts (hFOBs) and metastatic BC cells on a 3D bioprinted matrix incorporating calcium phosphate [43]. Co-culture of healthy and cancer cells on this scaffold altered cell morphology and proliferation and enhanced IL-8 secretion, which is a chemokine involved in inflammation, angiogenesis, and tumor progression. Despite the promising results, key challenges remain also for 3D bioprinting, particularly in optimizing scaffold porosity, mechanical strength, the spatial distribution of bioactive components to promote efficient cell integration, and the exploitation of patient-derived materials to mimic BM [43].

Organ-on-a-chip technologies further advance in vitro modeling by integrating microfluidic flow and multiple tissue-specific niches. Human bone marrow-on-a-chip platforms have successfully recapitulated essential features of the bone marrow microenvironment, including the endosteal, stromal, and vascular niches while supporting CD34^+^ hematopoietic stem cell maintenance and physiological signaling [44]. Similarly, osteosarcoma-on-a-chip systems have incorporated decellularized tumor ECM and bone marrow-derived extracellular vesicles into bioprinted scaffolds, combined with microfluidic perfusion, to recreate the complex tumor microenvironment and evaluate drug responses. These advanced platforms represent a new frontier in preclinical modeling of bone cancer metastasis and therapeutic testing. At the same time, the implementation of these approaches requires highly specialized instrumentation and expertise, which are often associated with substantial costs. Consequently, access to these technologies remains limited to a small number of research and clinical institutions. This underscores the need for alternative models that recapitulate the bone microenvironment while being amenable to broader scalability and dissemination.

Calcium phosphate-based biomaterials, including biomineralized polymers and porous HA scaffolds, are promising for this challenge for their excellent biomimicry and biocompatibility [45,46]. Numerous studies report in vitro assays of bone cements [45,47]. There are a variety of methods examining the biological performance of these materials, but most of the works reported evidence only on cancer cells. The challenge of co-culturing OBs, OCs, and their precursors is pivotal as well. Co-cultures are useful for evaluating responses to anticancer agents and for better mimicking the microenvironment [48,49]. In this work, we exploited highly biomimetic porous Sr-doped apatitic bone cements as a model to recapitulate the bone ECM and cellular components of the bone metastatic niche, especially considering the significant bioactivity and osteoinductivity character of its intrinsic physico-chemical features. In particular, previous studies demonstrated the successful osteogenic and osteointegrative capabilities of SrCPCs [26,50], including the multilevel role of strontium ions in rebalancing the bone metabolism, even in case of bone cancer [51]. The formulation obtained in this work demonstrated high biocompatibility with both bone and tumor cells [48]. The model allowed for an easy, tunable, and reproducible biomolecular characterization of cancer and bone cells in a step-by-step approach. SrCPCs were able to support cancer cells’ culture for 7 days, preserving their viability and morphology. Cancer cells grown on SrCPCs demonstrated a slower rate of proliferation than in monolayer culture, more closely reproducing the behavior of a developing in vivo tumor mass, as described by Liverani et al. [52]. Additionally, other authors have shown that collagen mineralization changes cancer phenotypes, including growth rate. Specifically, they demonstrated that MDA-MB-231 cell proliferation decreases when seeded on a mineralized material. This result is concordant with our observation, confirming that our preclinical platform recapitulates bone microenvironment and cancer cell–ECM crosstalk [53].

In addition to cancer cells, SrCPCs were assessed for the differentiation of mature bone cells from their progenitors. In particular, hMSCs and PBMCs were seeded on SrCPCs in presence of specific differentiating factors for OBs and OCs, respectively. After characterization of single cell-type cultures, we implemented the platform with a co-culture of cancer and bone cells in order to reproduce the bone metastatic niche. The fine optimization of our model enables tunable and flexible experiments to study the crosstalk in the bone metastatic niche, representing a step forward compared to previous works on similar cements [54], which only report results on the monoculture of cancer cells’ lines and without the exploitation of patient-derived materials. Some models mimicked the bone microenvironment in terms of ECM but completely lacked bone cells [55,56,57,58]. In particular, Peled et al. applied a bioactive 3D model able to mimic the endosteal bone microenvironment. They challenged the biomaterial with MDA-MB-231- and MCF7 BC-derived cells and demonstrated that close interactions between the cells and the biomaterial affect their proliferation rates and invasion properties. While this model guarantees to mimic cancer cell-ECM crosstalk, it lacks the main contribution of bone cells on the BM microenvironment [57].

Moreover, to improve the translatability of the experimental data, we exploited our model with patient-derived materials, namely PDEs and primary cell cultures [59,60,61,62,63].

In the literature, biological materials have been employed to study bone homeostasis and cancer progression [63]. It has been demonstrated that bone fragments maintain cell–matrix and cell–cell interactions in culture, allowing for the investigation of bone cells in their native environment [63]. Furthermore, ex vivo bone PDEs’ culture could be a more reliable model to resemble the human pathogenic metastasis condition and a useful tool to predict in vivo response to therapies [40,64]. Ex vivo assays using fresh rat or mouse bone have been developed successfully to test potential therapeutic drugs [46,47,65]. Compared to in vitro models, cultured bone fragments and PDEs better mimic the original tumor features, as they can preserve the in vivo 3D distribution and the innate proportion of osteocytes compared with other bone and stromal cells [64]. In this work, we used PDEs, both from metastatic and healthy peritumoral tissues, of two different patients as a proof of concept. To standardize the model as much as possible, the specimens were cut into different pieces of the same size, and only macroscopically homogeneous tissue types were chosen as technical replicates. Additionally, we isolated a primary cell line (BMET-3) from tumor tissue obtained from a third patient affected by BC-BM. To observe the impact of SrCPCs on patient-derived materials, we set up histology, viability, apoptosis, and necrosis assays. The overall results indicated a good biocompatibility of the SrCPCs, suggesting its potential future use as in vivo bone substitute. The main limitation of this type of experiment is the physiological loss of viability of some PDEs over time, which is more evident on day 7 of culture. However, as healthy cells appeared in better conditions than the tumor ones, we can hypothesize that necrosis did not depend solely on the ex vivo culture, but these necrotic areas could have also been present in the original tissue. However, this limitation could potentially be addressed by using a dynamic culture system that reproduces physiologically relevant culture conditions and by reducing the dimension of the PDEs to facilitate oxygen and nutrients diffusion. Moreover, to further increase the fidelity of our model, the components of the immune microenvironment should also be included. Although these are important aspects, in this paper, the use of the PDE model as a proof of concept satisfied our expectation and will be upgraded for future applications.

In conclusion, we leveraged an SrCPCs platform with bone mimicry property to develop an innovative, biocompatible, and tunable model of BM with tissue-like properties. Compared to the state of the art, our platform included the reciprocal interaction between cancer cells and specific bone cells, elucidating the multifaceted crosstalk that occurs at the metastatic site. This model holds promises for investigating the cellular and molecular mechanisms involved in BM and testing new potential therapeutic approaches. Moreover, we propose the use of SrCPCs to culture PDEs and the primary culture as a platform to investigate drug efficacy within the context of the entire tumor microenvironment architecture. Considering that this material already gave promising results in vivo [27], it may evolve, if conveniently designed and modified through a preliminary investigation on our in vitro platform, in other in vivo applications as a bone substitute for BM treatment.

## 5. Conclusions

In the present work, we studied an innovative preclinical platform-mimicking BM from BC. Co-culture of bone and cancer cells were optimized on bone-mimicking SrCPC to recreate the bone metastatic microenvironment. The material was also exploited with patient-derived materials achieving good results of viability. The platform demonstrated to be flexible, tunable, and provide reproducible results. This system can be customized according to specific needs, adding other different cell types, such as endothelial, stromal, and/or immune cells in static and/or dynamic culture systems, to recreate, in a physiologically relevant manner, the bone metastatic niche in the different phase of metastasis formation, i.e., in the early or in the late phase of this crucial process. It is noteworthy that this platform could be exploited as a tool for personalized medicine. In fact, further optimization would lead to the integration of patients’ cells, both cancer and stromal ones, recreating a proper patient-derived de-structured BM niche. Successively, a miniaturization process could potentially make this platform suitable for high throughput screening of drugs and innovative molecules. Altogether, in this work, we present a promising tool, both for research and clinical aims, that allow us to gain insight into new mechanisms of BM formation and study new therapeutic approaches in the context of a bone microenvironment.

## Figures and Tables

**Table 1:** List of assay catalog numbers of the primers used for real-time PCR for ex vivo samples.

Gene Symbol	Assay Catalog (Qiagen)
*TP53*	PPH00213A
*CASP9*	PPH00353A
*FOS*	PPH00094A
*BAX*	PPH00078A
*BBC3*	PPH02204A
*BCL2*	PPH00079A
*PARP1*	PPH00686A
*CASP3*	PPH00107A
*GAPDH*	PPH00150A

## Data Availability

The data that support the findings of this study are openly available in Zenodo Repository at https://doi.org/10.5281/zenodo.7781472 (accessed on 3 April 2023).

## Supplementary Materials

The following supporting information can be downloaded at https://www.mdpi.com/article/10.3390/jfb16060220/s1, Figure S1: Live/Dead on MDA-MB-231 in SrCPCs; Figure S2: Biocompatibility of SrCPCs on PDEs.
