# Supplementary material for: A Modular Biomimetic Preclinical Platform to Elucidate the Interaction Between Cancer Cells and the Bone Metastatic Niche
Source: J Funct Biomater. 2025 Jun 12;16(6):220. doi: 10.3390/jfb16060220 (PMC12194721; doi:10.3390/jfb16060220)
Supplement: Supplementary file 1 [file jfb-16-00220-s001.zip › jfb-3601766-supplementary.pdf]

Supplementary Material

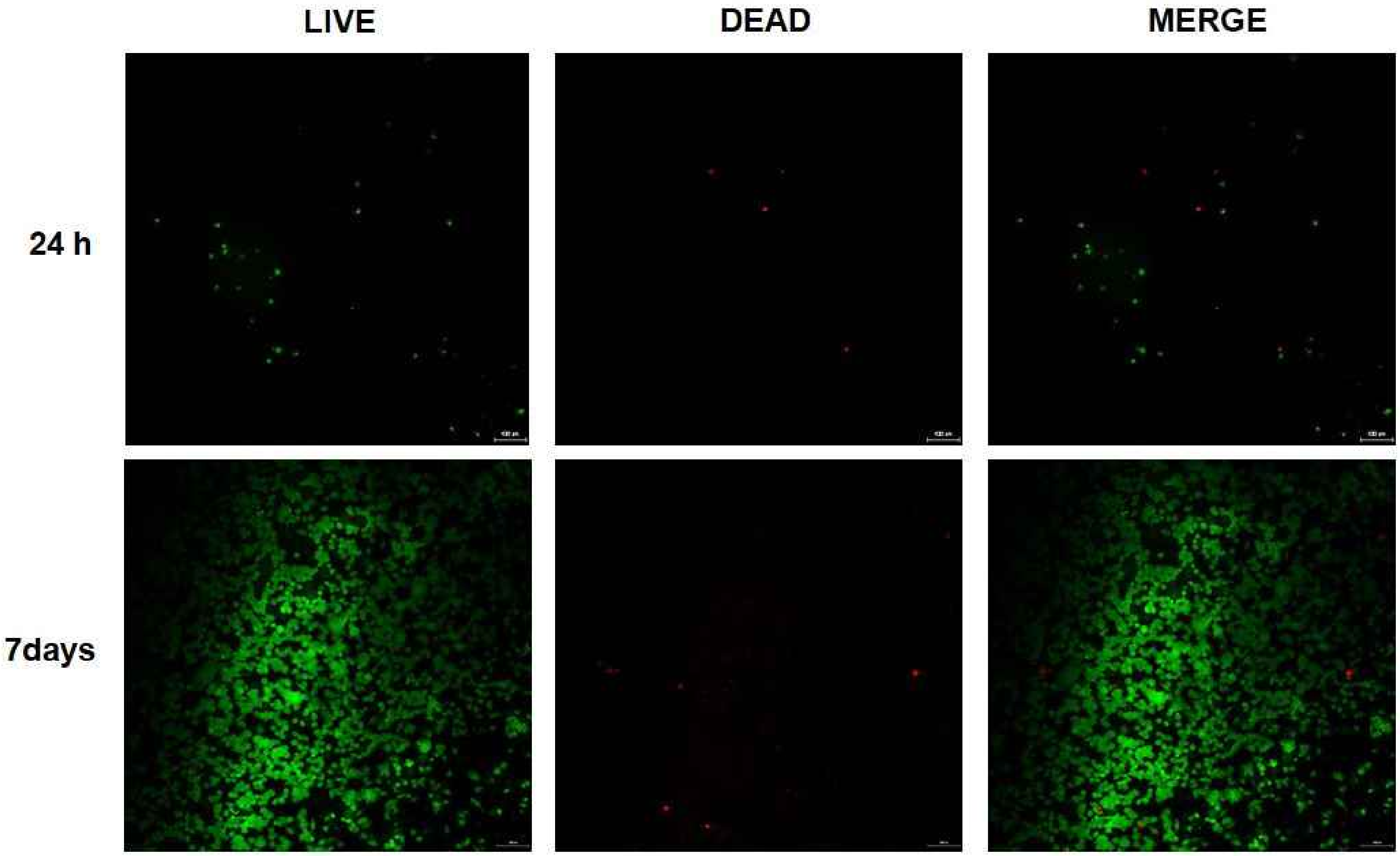

Figure S1. Live/dead on MDA-MB-231 in SrCPCs. Confocal microscopy images of MDA-MB-231 at 20X magnification of live/dead staining at day 1 (24h) and day 7. In the first column live cells were stained in green; in the second column dead cells were stained in red; in the third column the 2 stainings were merged.

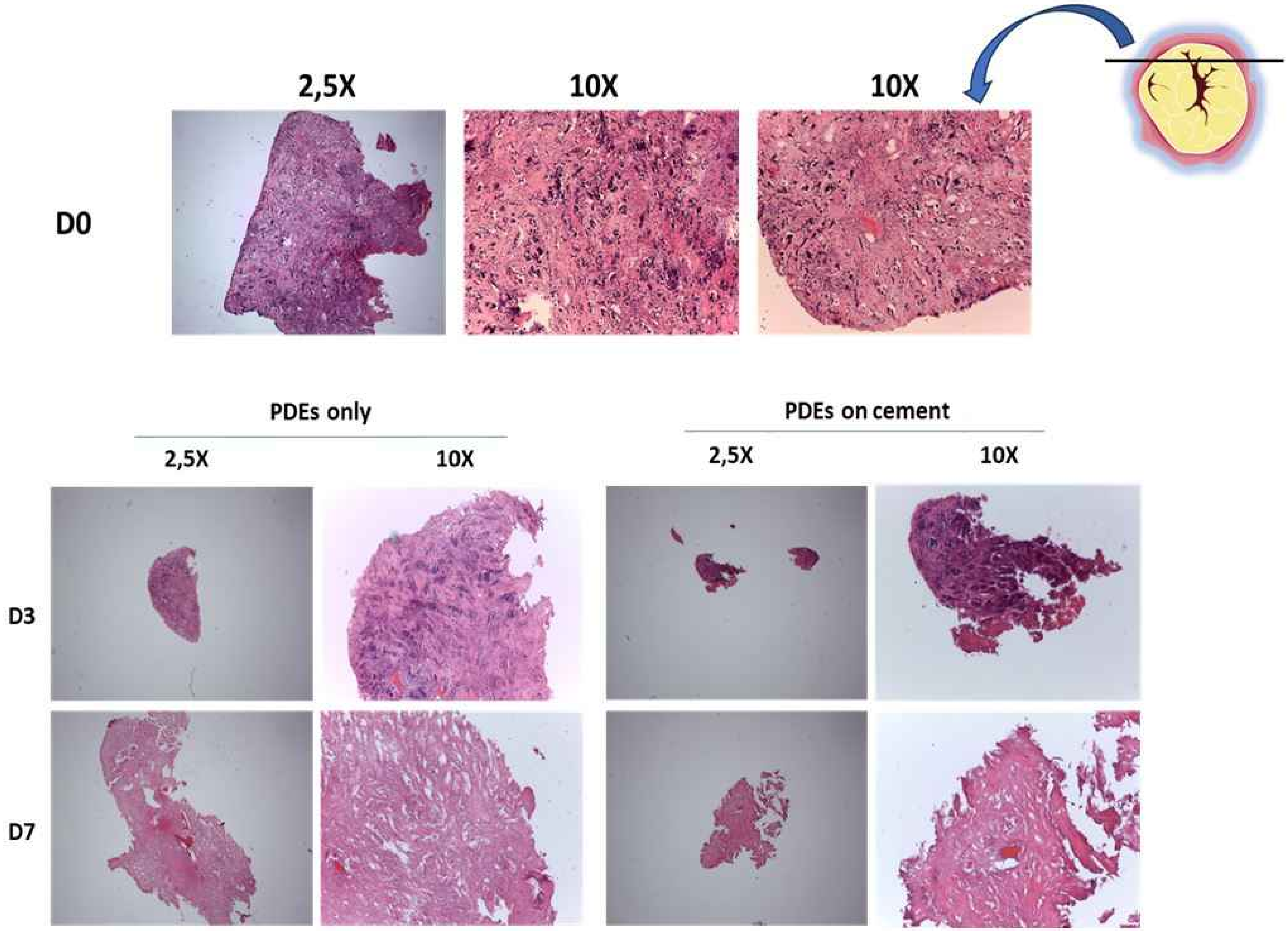

Figure S2. Biocompatibility of SrCPCs on PDEs. Representative pictures of the H&E staining of the external region of the PDEs. Upper panel) baseline tissue architecture and viability of Patient 1 material before the culture set up (Day 0); Bottom left panel) Representative picture of PDEs only after 3 and 7 days of culture; Bottom right panel) Representative picture of PDEs cultured on SrCPCs after 3 and 7 days of culture.
